# Prevalence of spina bifida across the lifespan in the USA

**DOI:** 10.1111/dmcn.70161

**Published:** 2026-01-29

**Authors:** Julie Bershadsky, Catharine Riley, Sandra L. Pettingell, Sheryl A. Larson, Jennifer Hall-Lande, Libby Hallas, Tiebin Liu, Jennita Reefhuis

**Affiliations:** 1 Institute on Community Integration, University of Minnesota, Minneapolis, MN, USA; 2 Centers for Disease Control and Prevention, Atlanta, GA, USA

## Abstract

**Aim:**

To estimate the prevalence of spina bifida across the lifespan, the number of people with spina bifida by state, and variations in spina bifida prevalence by insurance type, state, and selected demographics.

**Method:**

This was an observational study that used administrative insurance data. We used three public-payer data sets (Medicaid Fee for Service, Medicare, and Medicare Advantage) and one large employer-sponsored insurance database (Health Care Cost Institute [HCCI]) to estimate all-age prevalence of spina bifida in the USA. Claims data from 2017 to 2019 were used to identify spina bifida based on International Classification of Diseases, 10th Revision codes.

**Results:**

The overall prevalence estimate of spina bifida was 3.20 per 10 000 (7.03 for Medicaid, 5.03 for Medicare, 1.72 for HCCI) and varied by demographics. We estimate at least 92 551 people in the USA live with spina bifida.

**Interpretation:**

Most spina bifida prevalence estimates focus on birth prevalence. This study demonstrates that prevalence estimates across all ages throughout the lifespan can be generated using multiple insurance data sources.

Having a robust estimate of prevalence across the lifespan for spina bifida is foundational to supporting public health and clinical research. Spina bifida is a neural tube defect present at birth that occurs when the neural tube does not close all the way. Several published studies have estimated the birth prevalence of spina bifida. A meta-analysis of 129 birth prevalence articles reported spina bifida prevalence estimates of 3.59 across 29 postfolic acid food fortification North American studies.^1^ National Birth Defects Prevention Network data from 31 states/geographic regions for 2010 to 2014 reported birth prevalence of 3.63 per 10 000 for spina bifida adjusted for maternal ethnicity.^2^ This was a decrease compared to National Birth Defects Prevention Network estimates from before the USA mandated folic acid supplementation (4.3–6.5 per 10 000, 1999–2011).^3^ While birth prevalence estimates identify the rate at which new cases of spina bifida emerge, they do not provide information about changes in prevalence rates across the lifespan. Furthermore, while the National Birth Defects Prevention Network covers 31 states, administrative data sets with coverage across all states covering both public and private sectors can supplement and enhance our understanding of spina bifida.

Two previous studies used commercial insurance claims data to estimate the prevalence of spina bifida. An analysis of MarketScan commercial insurance claims data, representing employer-sponsored health insurance, estimated the prevalence of spina bifida (ages birth to 64 years) to be 21.8 per 100 000 for the year 2000, 21.2 for 2001, 20.7 for 2002, 19.5 for 2003, and for a weighted 4-year average prevalence rate of 20.6 per 100 000.^4,5^ Differences across studies in estimates of spina bifida prevalence may be due to factors such as study date, ages included, methodologies used, and whether the data used were collected before or after folic acid fortification in the USA.

There are no current estimates of the number of people of all ages living with spina bifida in the USA generated from nationally representative data. Our study aimed to estimate prevalence rates for spina bifida across the lifespan using administrative data from multiple payer types to estimate the total number of people living with spina bifida in the USA. The study was reviewed by the University of Minnesota Institutional Review Board and ruled exempt.

Public-payer administrative data sets include people who participate in the public-payer insurance program. Medicaid and the Children's Health Insurance Program (covering 19.8% of the USA population) and Medicare (covering 18.1% of the USA population) are the largest providers of publicly funded health and long-term care in the USA (https://www.census.gov/data/tables/time-series/demo/health-insurance/acs-hi.2019.html#list-tab-776654388). Participants may be enrolled in a plan providing fee-for-service care, a managed care program, or may receive care for both coverage types (e.g. acute care services through a managed care organization; long-term supports and services through a fee-for-service plan). Participation is limited to people who qualify based on age, income, assets, and presence of a qualifying disability or condition.

Commercial insurance may cover individuals with or without qualifying spouses and dependents. Eligibility criteria limit participation in group plans to households with a member employed by an enrolled organization, individuals who are part of a participating group, or to people who enroll individually. An estimated 67.4% of the USA population were covered by commercial insurance in 2019 (https://www.census.gov/data/tables/time-series/demo/health-insurance/acs-hi.2019.html#list-tab-776654388). This analysis used health insurance claims from employer-sponsored insurance (ESI), Medicaid, and Medicare to estimate the prevalence of spina bifida.

## METHOD

### Data sources

#### Employer-sponsored insurance

Health Care Cost Institute (HCCI) is an independent, non-profit research institute that manages private insurance claims data from four health insurance companies (Aetna, Humana, Kaiser Permanente, and Blue Health Intelligence; https://healthcostinstitute.org/about-hcci). The database represents approximately 40% of all individuals aged 18 to 64 years in the USA who are privately insured through employer-sponsored plans and their dependent family members. Access was obtained to all claims data on all covered individuals over an approximately 10-year period. Our study used HCCI inpatient, outpatient, and physician claims from 2017 to 2019.

#### Medicaid and Medicare

Data procured from the Research Data Assistance Center/Chronic Conditions Warehouse included all Medicaid and Medicare (covered in Medicare Fee-for-Service or Medicare Advantage) beneficiaries in 2019 (the latest data available). Medicaid coverage includes low-income individuals, children, pregnant females, the elderly, and individuals with disabilities. Medicare coverage is primarily for adults aged 65 years and older, but younger individuals with disabilities can also be covered.

Claims data requested from Research Data Assistance Center were limited to those minimally necessary for the study. Three years of Medicaid claims data (2017–2019) were used to identify enrollees with at least one International Classification of Diseases, 10th Revision (ICD-10) spina bifida diagnosis code in those 3 years and request all claims data for those enrollees. Data requested included Transformed Medicaid Statistical Information System (T-MSIS) analytic file demographic and eligibility data for all Medicaid beneficiaries in 2019 as well as inpatient hospital, long-term care, and other services data from 2017 to 2019 (files that capture ICD-10 diagnostic codes).

For Medicare, we requested the master beneficiary summary file and multiple claim files. For Medicare Fee-for-Service, this included all inpatient, outpatient, skilled nursing facilities, hospice, home health agency, carrier, and durable medical equipment data. For Medicare Advantage/Part C, this included all encounter data contained in inpatient, outpatient, specialized nursing facility, home health, carrier, and durable medical equipment data sets.

#### Spina bifida identification

The Research Data Assistance Center/Chronic Conditions Warehouse published an algorithm for identifying ‘spina bifida and other congenital anomalies of the nervous system’ using diagnostic or procedure codes (https://www2.ccwdata.org/web/guest/condition-categories-other (data is only available to US-based users)). We modified this algorithm to exclude individuals who did not have spina bifida. Individuals with spina bifida were identified using ICD-10 diagnostic codes Q05.xx. The complete list of codes can be found in Table S1. To be classified as having spina bifida, enrollees had to have at least one distinct inpatient claim (ESI), inpatient or long-term care claim (Medicaid), inpatient or skilled nursing facility or hospice claim (Medicare), or two distinct other claims with a spina bifida ICD-10 code from 1st January 2017 to 31st December 2019.

It should be noted that estimating prevalence through case ascertainment with ICD codes may be affected by differences in provider coding practices. For instance, providers may choose to record spina bifida as an underlying condition during preventive health screenings, but they are not obligated to do so. Consequently, the actual prevalence may be higher.

#### Inclusion and exclusion criteria for estimating prevalence

Individuals covered under ESI, Medicaid, or Medicare were included if they had at least 1 day of active enrollment in every month of 2019. The decision to include only those with 12 months active enrollment was based on previous studies that required 12 months of continuous coverage.^5-7^ An additional inclusion requirement for ESI was being under 65 years of age, since Medicare is the primary insurance coverage for people aged 65 years and over. Individuals who were not insured were not captured and therefore excluded from analysis.

### Analysis

All analyses were conducted inside secure, virtual environments, NORC Data Enclave hosted by the University of Chicago for HCCI data and Centers for Medicare and Medicaid Services' Chronic Conditions Warehouse Virtual Research Data Center for Medicaid and Medicare data.

#### Prevalence calculation

Prevalence rate estimates were calculated as the number of individuals with spina bifida per 10 000. Separate prevalence rate estimates were calculated for ESI, Medicaid, and Medicare. Rates are presented for the USA overall and by state.

#### Confidence intervals

Prevalence estimates were calculated with 95% confidence intervals (CIs) to quantify the precision of the estimates. Wilson score interval 95% CIs were computed for overall and state spina bifida prevalence estimates using a proportion CI calculator (https://www.statskingdom.com/proportion-confidence-interval-calculator.html). Each calculation was multiplied by 10 000 as prevalence estimates were per 10 000 insured individuals.

#### National extrapolation

Prevalence estimates from each insurance type were extrapolated to estimate national prevalence. Prevalence estimates were applied to the number of people in corresponding insurance programs in the USA. We used 2019 estimates from the American Community Survey for the overall numbers of people in the USA in private insurance (largely comprised of ESI), Medicaid, and Medicare (https://www.census.gov/data/tables/time-series/demo/health-insurance/acs-hi.2019.html#list-tab-776654388). The American Community Survey also provides the number of people who are covered by more than one type of insurance (i.e. for each combination of dual coverage).

We developed an original four-step extrapolation method and applied it in this paper.

(1) As Medicare is generally the primary payer (i.e. Medicare pays first if a beneficiary has more than one type of health insurance coverage), we first applied the Medicare-based estimated prevalence to the American Community Survey-derived total Medicare-insured population in 2019.


$$N_{1}=\frac{\left(Medicare\;prevalence)\times (Medicare\;population\right)}{10\;000}$$

(2) When a beneficiary has both Medicare and ESI, ESI generally pays second. We therefore subtracted the dual Medicare-ESI population from the American Community Survey ESI population estimates and applied our private insurance based estimated prevalence to the non-overlapping ESI population.


$$N_{2}=\frac{\left(ESI\;prevalence)\times (ESI\;population-ESI\&Medicare\;dual\;population\right)}{10\;000}$$

(3) Medicaid is generally the last payer. We applied the Medicaid-based estimated prevalence to the non-overlapping Medicaid insurance population in 2019.


$$N_{3}=\frac{\left(Medicare\;prevalence)\times (Medicaid\;population-Medicaid\&Medicare\;dual\;population-ESI\&Medicare\;dual\;population\right)}{10\;000}$$

(4) The final step was to add steps 1 to 3 to arrive at the total estimated number of people with spina bifida in the USA covered by these three insurance types.


$$N_{\text{Total Extrapolated}}=N1+N2+N3$$

This calculation does not include the approximately 12% of the USA population that is covered by TRICARE or Veterans Affairs or is uninsured. Therefore, the extrapolated numbers and national prevalence estimates are likely an undercount.

#### Algorithm validation

Algorithm validation was conducted to confirm and validate our methodological approach and assess the impact of potential misclassification and missing claims on overall prevalence estimates. We used HCCI data from 2018 to 2020 to conduct the validation and compare our spina bifida prevalence rate to the prevalence rate calculated using data from 2018 to 2020.

## RESULTS

Spina bifida prevalence estimates were computed for each insurance type and extrapolated per 10 000 insured individuals in the USA (Table 1). There were 35 581 831 ESI enrollees under 65 years of age with 12 months of active enrollment in 2019. Of those, 6116 enrollees were identified as having spina bifida. The resulting ESI prevalence estimate of spina bifida was 1.72 per 10 000 enrollees (Table 1).

There were 61 345 530 Medicaid enrollees with at least 1 day of active enrollment in every month of 2019, including 43 125 identified as having spina bifida. The resulting Medicaid prevalence estimate of spina bifida was 7.03 per 10 000 enrollees.

There were 58 659 427 enrollees in Medicare (Medicare Fee-for-Service, Medicare Advantage, or both) who had 12 months of active enrollment in every month of 2019. There were 29 487 Medicare enrollees identified as having spina bifida. The resulting estimated prevalence among Medicare enrollees was 5.03 per 10 000 enrollees.

The national prevalence estimate for spina bifida was 3.20 (3.18–3.22) per 10 000 insured individuals in the USA (Table 1). The estimated number of individuals living with spina bifida was at least 92 551.

We also calculated prevalence estimates and estimated number of individuals living with spina bifida for each state, as well as by sex, age, and ethnicity.

The estimated prevalence rate of spina bifida ranged from a low of 1.33 per 10 000 insured individuals in Hawaii to a high of 5.25 per 10 000 in West Virginia (Table 2).

Prevalence estimates of spina bifida by sex, age, and ethnicity within insurance type are shown in Table 3. Estimates varied by insurance type. Spina bifida rates were higher for male Medicaid enrollees but were higher for female ESI and Medicare enrollees. Spina bifida prevalence estimates by age also varied by insurance type. Amongst ESI and Medicare enrollees, prevalence estimates were highest for younger people and became progressively lower as age increased. Spina bifida prevalence estimates for Medicaid were highest for adults aged 18 to 44 years.

Ethnicity data were not available for ESI enrollees. Among Medicaid enrollees, highest spina bifida prevalence rates were estimated for White, non-Hispanic enrollees, followed by Hispanic enrollees and American Indian or Alaskan Native enrollees. Amongst Medicare enrollees, highest spina bifida prevalence rates were estimated for Hispanic enrollees followed by American Indian or Alaskan Native enrollees, then by White Non-Hispanic enrollees. Table S2 presents sex, age, and ethnicity variations by type of insurance in the USA population at large.

### Algorithm validation

Using HCCI data from 2018 to 2020, algorithm validation was conducted and found prevalence rates comparable to those based on data from 2017 to 2019. Prevalence rate of spina bifida using data from 2018 to 2020 was 1.68 per 10 000 enrollees compared to 1.72 using data from 2017 to 2019. Results were also similar for demographic groups (data not shown, but available on request).

### Sensitivity analysis

In order to understand the potential effects of our data not accounting for the 12% of the USA population without health insurance or with insurance through TRICARE or Veterans Affairs, we performed a simple sensitivity analysis. The true overall prevalence estimate (i.e. the prevalence in 100% of the data, if we had all of it) is going to be somewhat lower than the current estimate of 3.20 per 10 000 if the missing 12% had no or lower spina bifida prevalence (e.g. 0 per 10 000), and somewhat higher if the missing 12% had higher spina bifida prevalence. We can simulate the effect of these scenarios using a simple formula as follows (x being the unknown prevalence in the 12% of non-covered population):

$$\text{True prevalence}=(3.20^{*}\;0.88)+(0.12^{*}\;x)$$

Table 4 presents the results of this sensitivity analysis. If we assume that the missing prevalence in the 12% is 0 per 10 000, then the true overall prevalence is lower by 12% and is 2.82 per 10 000. If the prevalence in the missing 12% is same as in Medicaid, then the true overall prevalence is higher by 14% and is 3.66 per 10 000. If the prevalence in the missing 12% is even higher and is three times than estimated, then the true overall prevalence is 3.97 per 10 000.

## DISCUSSION

We estimate that there were at least 92 551 people of all ages in the USA with spina bifida in 2019. The national prevalence of spina bifida for people with ESI, Medicaid, or Medicare was estimated to be 3.20 per 10 000 insured population. This estimate varied by insurance type (ESI, 1.72 per 10 000; Medicaid, 7.03 per 10 000; Medicare, 5.03 per 10 000) and was lower than the estimates of 3.59 to 3.63 per 10 000 reported in previous birth prevalence studies.^2,3^

Variations in prevalence rates by state, insurance type, and age were expected given the complexity of the USA healthcare insurance infrastructure. Individual characteristics vary by insurance type (Table S2) and prevalence estimates ranged the most by insurance type. ESI is primarily used by adults who are employed and have an income above 138% of the federal poverty level. ESI generally offers family plans, so spouses and dependents up to age 25 years are often covered by the same insurance. Medicare enrollees are mostly 65 years or older, no longer employed, and have family incomes above 138% of the federal poverty level.^8^ Medicaid and the Children's Health Insurance Program enrollees are children and working-age adults, most of whom are not employed and have family incomes below 138% of the federal poverty level. Differences in spina bifida rates by age and ethnicity are likely related to different patterns of insurance enrollment. However, differences in spina bifida prevalence rates by sex for the three types of insurance do not appear to be related to differences in insurance coverage patterns since there were more females than males with spina bifida in all three insurance types. Those differences may be due to factors not examined in this study.

There were several limitations to this study. First, we did not have data for the 12% of the USA population without health insurance or with insurance through TRICARE or Veterans Affairs.^9^ The prevalence of spina bifida amongst people who are unemployed or who are covered through TRICARE or Veterans Affairs may differ from the prevalence rates in the types of insurance included in this study. Second, the HCCI ESI database covers only 40% of the total population with ESI. Differences between people enrolled in ESI plans not included in the HCCI database versus those covered by HCCI are not known. Third, ICD codes are used primarily for billing purposes. Using ICD codes for case ascertainment to estimate prevalence could be impacted by variations in provider coding practices across all data sources. Providers can choose to code spina bifida as an underlying condition if a patient is seen for preventative health screenings, for instance, but it is not required. Therefore, true prevalence may be higher.

We used all ICD codes recorded for enrollees over a 3-year period to maximize case ascertainment. Previous studies have shown that using 3 years rather than 2 years of claims data increased the likelihood that a diagnosis of spina bifida would be in the electronic record for persons with spina bifida.^1,5^

We hoped to be able to stratify the national prevalence estimates by ethnicity. However, HCCI records do not include ethnicity data and the quality of ethnicity data in the Medicaid and Medicare data sources vary by state. We found variations in prevalence by state, age, and sex across insurance types. However, more information is needed to assess the impact of differing eligibility criteria across states and insurance types.

Strengths of this analysis include the large sample size of private insurance enrollees, and the inclusion of all Medicaid and Medicare enrollees, providing robust and generalizable estimates of spina bifida prevalence. Furthermore, spina bifida is a condition for which a stable set of specific ICD codes have been used in the clinical setting for many years, including the 3 years in the study. Finally, this is the first study reporting all-age spina bifida prevalence rate estimates by state. It supplements the information provided by birth prevalence studies by examining prevalence across the lifespan and differences by insurance type, age, sex, and ethnicity.

Our study illustrates the feasibility of assessing prevalence of rare conditions using large claims databases. It shows the benefit of assessing prevalence across both the private and public insurance sectors since estimates varied widely across the administrative data sets. Furthermore, most previous spina bifida prevalence estimates have been based on birth prevalence rather than across the lifespan. Life expectancy for people with spina bifida has increased over recent decades, providing hope for parents whose infant is diagnosed with spina bifida. Having prevalence estimates for people of all ages, geographies, and insurance types gives public health agencies and policymakers a better sense of the size and needs of the spina bifida community. This could enable improved planning for public health activities as well as research and clinical services to optimize the health and well-being of those living with spina bifida.

## Supplementary Material

SUP 1- Bershadsky - Prevalence of spina bifida across the lifespan in the USA

SUP 2- Bershadsky - Prevalence of spina bifida across the lifespan in the USA

The following additional material may be found online:

**Table S1** ICD-10 spina bifida diagnosis codes

**Table S2** Demographic characteristics of people in the USA by health insurance type

## Figures and Tables

**TABLE 1:** Prevalence estimates for spina bifida by insurance type, per 10 000 insurance enrollees and overall insured USA population, 2019.

Insurance type	Identified cases	Denominator	Prevalence per 10 000 (95% CI)
Employer-sponsored insurance	6116	35 581 831	1.72 (1.68–1.76)
Medicaid	43 125	61 345 530	7.03 (6.96–7.10)
Medicare	29 487	58 659 427	5.03 (5.01–5.13)
**Overall insured USA population**			**3.20 (3.18–3.22)**

Abbreviation: CI, confidence interval.

**TABLE 2:** Estimates of spina bifida prevalence (per 10 000 insured population) and number of individuals with spina bifida by state, extrapolated from employer-sponsored insurance, Medicaid, and Medicare, 2019.

State	Spina bifida prevalence (95% CI)	Estimated number of people with spina bifida (95% CI)
Alabama	3.81 (3.63–4.00)	1627 (1368–1963)
Alaska	2.60 (2.23–3.04)	159 (115–221)
Arizona	3.29 (3.29–3.43)	2059 (1855–2288)
Arkansas	4.16 (3.92–4.42)	1100 (954 – 1296)
California	2.43 (2.38–2.48)	8652 (8152–9194)
Colorado	2.60 (2.47–2.75)	1339 (1140–1585)
Connecticut	2.75 (2.57–2.93)	895 (766–1053)
Delaware	3.17 (2.82–3.57)	278 (217–356)
District of Columbia	1.44 (1.18–1.76)	95 (61–153)
Florida	2.86 (2.78–2.94)	5163 (4831–5525)
Georgia	3.19 (3.08–3.31)	2828 (2567–3121)
Hawaii	1.33 (1.15–1.54)	171 (103–317)
Idaho	3.90 (3.60–4.22)	604 (495–739)
Illinois	2.85 (2.75–2.95)	3263 (3009–3540)
Indiana	4.01 (3.85–4.17)	2395 (2149–2673)
Iowa	4.28 (4.05–4.52)	1246 (1047–1492)
Kansas	3.60 (3.37–3.84)	921 (798–1063)
Kentucky	4.27 (4.07–4.48)	1725 (1534–1946)
Louisiana	4.08 (3.89–4.29)	1663 (1460–1857)
Maine	3.34 (3.03–3.69)	398 (317–515)
Maryland	2.61 (2.48–2.75)	1441 (1275–1629)
Massachusetts	2.90 (2.78–3.04)	1892 (1696–2116)
Michigan	3.63 (3.51–3.76)	3327 (3046–3638)
Minnesota	3.37 (3.22–3.53)	1773 (1543–2053)
Mississippi	4.13 (3.89–4.39)	1025 (871–1215)
Missouri	3.95 (3.79–4.12)	2115 (1896–2364)
Montana	3.01 (2.68–3.38)	286 (207–405)
Nebraska	3.86 (3.58–4.17)	667 (560–796)
Nevada	2.38 (2.20–2.57)	633 (498–818)
New Hampshire	3.73 (3.41–4.09)	465 (361 – 603)
New Jersey	2.55 (2.45–2.67)	2044 (1847–2262)
New Mexico	3.45 (3.20–3.74)	629 (507–798)
New York	2.67 (2.60–2.75)	4800 (4465–5166)
North Carolina	3.40 (3.28–3.52)	3049 (2798–3326)
North Dakota	3.41 (3.00–3.88)	233 (144–398)
Ohio	4.03 (3.91–4.15)	4284 (3934–4675)
Oklahoma	3.96 (3.76–4.19)	1296 (1129–1492)
Oregon	3.24 (3.07–3.43)	1238 (1030–1507)
Pennsylvania	3.83 (3.72–3.94)	4471 (4189–4775)
Rhode Island	3.48 (3.13–3.87)	343 (250–483)
South Carolina	2.86 (2.71–3.02)	1265 (1105–1456)
South Dakota	3.78 (3.37–4.24)	291 (189–464)
Tennessee	3.76 (3.61–3.92)	2234 (2030–2460)
Texas	3.91 (3.83–4.00)	8997 (8534–9489)
Utah	3.84 (3.62–4.08)	1095 (892–1352)
Vermont	3.76 (3.30–4.30)	219 (157–329)
Virginia	2.90 (2.78–3.02)	2184 (1951–2450)
Washington	2.54 (2.43–2.67)	1758 (1568–1973)
West Virginia	5.25 (4.91–5.62)	846 (725–992)
Wisconsin	3.59 (3.43–3.75)	1918 (1689–2188)
Wyoming	2.63 (2.21–3.12)	129 (78–223)

CI calculated using: https://www.statskingdom.com/proportion-confidence-interval-calculator.html

Abbreviation: CI, confidence interval.

**TABLE 3:** Spina bifida prevalence rate estimates per 10 000 enrollees by sex, age, and ethnicity, and by health insurance type, 2019.

	Spina bifida prevalence per 10 000 enrollees		
Characteristic	ESI^a^	Medicaid	Medicare
**Sex**			
Male	1.48	7.14	4.77
Female	1.95	6.94	5.24
**Age in years**			
0 to 17	2.96	5.74	108.51^b^
18 to 24	2.17	10.65	77.99
25 to 34	1.83	12.62	69.82
35 to 44	1.38	11.02	54.42
45 to 54	1.02	8.24	29.31
55 to 64	0.79	4.01	9.85
65 and up	--	1.54	1.80
**Ethnicity**			
American Indian or Alaskan Native, non-Hispanic	--	5.63	8.94
Asian, Non-Hispanic	--	1.93	1.65
Black, Non-Hispanic	--	3.88	4.40
Hispanic	--	6.14	9.57
White, Non-Hispanic	--	9.20	5.10

a ESI data for individuals over 65 years of age were excluded and no ethnicity data were available for ESI enrollees.

b Estimate is based on a very small number of enrollees.

Abbreviation: ESI, employer-sponsored insurance.

**TABLE 4:** Sensitivity analysis: the ‘missing’ 12% and its effect on estimated prevalence.

Assumption	Assumed prevalence in ‘missing’ 12% of population (per 10 000)	Overall prevalence	Percent change from current estimated prevalence
No spina bifida	0.00	2.82	−12%
Same as commercial insurance population	1.72	3.02	−6%
Same as current estimated prevalence	3.20	3.20	0%
Same as Medicare population	5.03	3.42	+7%
2x the current estimated prevalence	6.40	3.58	+12%
Same as Medicaid population	7.03	3.66	+14%
3x current estimated prevalence	9.60	3.97	+24%

## Data Availability

The data that support the findings of this study are available from the corresponding author upon reasonable request.

## What this paper adds

- Spina bifida prevalence is estimated to be 3.20 per 10 000 people in the USA.
- Spina bifida prevalence estimates differ across public and private insurance data sources.
- Spina bifida prevalence estimates differ geographically and across age, sex, and ethnicity.

## Abbreviations:

ESI: employer-sponsored insurance
HCCI: Health Care Cost Institute
