# Supplementary material for: Prevalence of spina bifida across the lifespan in the USA
Source: Dev Med Child Neurol. Author manuscript; Available in PMC 2026 Jun 25. (PMC13292501; doi:10.1111/dmcn.70161)
Supplement: SUP 1- Bershadsky - Prevalence of spina bifida across the lifespan in the USA [file NIHMS2176000-supplement-SUP_1-_Bershadsky_-_Prevalence_of_spina_bifida_across_the_lifespan_in_the_USA.docx]

Table S1. ICD-10 Spina Bifida Diagnosis (Dx) Codes

| **ICD-10 Dx Code** | **Definition of ICD-10 Dx Code** |
| --- | --- |
| Q05 | Spina bifida |
| Q05.0 | Cervical spina bifida with hydrocephalus |
| Q05.1 | Thoracic spina bifida with hydrocephalus |
| Q05.2 | Lumbar spina bifida with hydrocephalus |
| Q05.3 | Sacral spina bifida with hydrocephalus |
| Q05.4 | Unspecified spina bifida with hydrocephalus |
| Q05.5 | Cervical spina bifida without hydrocephalus |
| Q05.6 | Thoracic spina bifida without hydrocephalus |
| Q05.7 | Lumbar spina bifida without hydrocephalus |
| Q05.8 | Sacral spina bifida without hydrocephalus |
| Q05.9 | Spina bifida, unspecified |

*Note.* ICD = International Classification of Diseases.
