# Supplementary material for: Prevalence of spina bifida across the lifespan in the USA
Source: Dev Med Child Neurol. Author manuscript; Available in PMC 2026 Jun 25. (PMC13292501; doi:10.1111/dmcn.70161)
Supplement: SUP 2- Bershadsky - Prevalence of spina bifida across the lifespan in the USA [file NIHMS2176000-supplement-SUP_2-_Bershadsky_-_Prevalence_of_spina_bifida_across_the_lifespan_in_the_USA.docx]

Table S2. Demographic Characteristics of People in the U.S. by Health Insurance Type

| **Characteristic** | **Private Insurance** | **Medicaid or CHIP** | **Medicare** |
| --- | --- | --- | --- |
| **Age in years** |  |  |  |
| 0-18 | 20.9% | 48.8% | < 0.3% |
| 19-64 | 65.6% | 44.1% | 12.3% |
| 65 or older | 13.5% | 7.2% | 87.6% |
| **Sex** |  |  |  |
| Male | 49.1% | 45.2% | 44.9% |
| Female | 50.9% | 54.8% | 55.1% |
| **Race/Ethnicity** |  |  |  |
| Hispanic | 13.1% | 30.5% | 9.0% |
| White, non-Hispanic | 68.3% | 39.5% | 74.3% |
| Black, non-Hispanic | 9.3% | 19.3% | 10.6% |
| American Indian or Alaska Native, non-Hispanic | 0.3% | <0.3% | 0.5% |
| Asian, non-Hispanic | 6.5% | 5.1% | 4.2% |
| Other, non-Hispanic | 2.5% | 4.1% | 1.4% |
| **Employment/Income** |  |  |  |
| Family income less than 138% of Federal Poverty Level | 6.2% | 53.2% | 18.6% |
| Currently employed | 74.0% | 39.6% | 15.8% |

*Note. CHIP = Children’s Health Insurance Program. The data source is MACPAC, 2022; Exhibit 2; MACPAC (2022). MACStats: Medicaid and CHIP Data Book. https://www.macpac.gov/publication/macstats-medicaid-and-chip-data-book-2/*
